# Structural and Emulsifying Properties of Soybean Protein Isolate–Sodium Alginate Conjugates under High Hydrostatic Pressure

**DOI:** 10.3390/foods10112829

**Published:** 2021-11-17

**Authors:** Zihuan Wang, Shaoying Gong, Yucong Wang, Danyi Liu, Jianchun Han

**Affiliations:** 1 College of Food Science, Northeast Agricultural University, Harbin 150030, China; wangzihuan0311@163.com (Z.W.); gong_shaoying@126.com (S.G.); wangyucong1115@163.com (Y.W.); 2 Heilongjiang Green Food Science Research Institute, Harbin 150030, China

**Keywords:** high hydrostatic pressure, soybean protein isolate, sodium alginate, maillard reaction, emulsifying property, protein structure, soybean protein isolate–sodium alginate conjugates

## Abstract

Soybean protein isolate (SPI) is a kind of plant derived protein with high nutritional value, but it is underutilized due to its structural limitations and poor functionalities. This study aimed to investigate the effects of high hydrostatic pressure (HHP) treatment on SPI and sodium alginate (SA) conjugates prepared through the Maillard reaction. The physicochemical properties of the conjugate synthesized under 200 MPa at 60 °C for 24 h (SPI–SA–200) were compared with those of the conjugate synthesized under atmospheric pressure (SPI–SA–0.1), SPI-SA mixture, and SPI. The HHP (200 MPa) significantly hindered the Maillard reaction. This effect was confirmed by performing SDS-PAGE. The alterations in the secondary structures, such as α-helices, were analyzed using circular dichroism spectroscopy and the fluorescence intensity was determined. Emulsifying activity and stability indices of SPI-SA-200 increased by 33.56% and 31.96% respectively in comparison with the SPI–SA–0.1 conjugate. Furthermore, reduced particle sizes (356.18 nm), enhanced zeta potential (‒40.95 mV), and homogeneous droplet sizes were observed for the SPI-SA-200 emulsion. The present study details a practical method to prepare desirable emulsifiers for food processing by controlling the Maillard reaction and improving the functionality of SPI.

## 1. Introduction

Soybean protein isolate (SPI) is an abundant plant protein with a protein content of approximately 90% and a variety of essential amino acids that exhibit excellent functional properties and high nutritional content [1,2]. Because SPI contains both hydrophilic and hydrophobic groups, it is commonly employed as an emulsifier in food preparation [3]. Previous research has established that SPI could exist and reduce the surface tension at the oil–water interface in the emulsion system [4,5]. Moreover, the electrostatic repulsion and steric hindrance between the SPI molecules could effectively prevent flocculation caused by the aggregation of droplets [6]. However, the structure of SPI is so dense that the functional properties are poor and the emulsifying properties of SPI are significantly susceptible to the external environment, which can induce coalescence and creaming [7,8].

As natural biopolymers, polysaccharides are widely employed to enhance the emulsifying properties of protein emulsions, since they improve the interfacial and rheological properties by increasing the viscosity of the continuous phase [9]. Sodium alginate (SA) is a brown-algae-derived anionic polysaccharide composed of units of (1,4)-D-mannuronic acid and (1,4)-L-guluronic acid. Owing to its viscous, steric, and electrostatic properties, SA exhibits excellent characteristics in terms of emulsion formation and stabilization in aqueous solutions [10,11].

Proteins and polysaccharides are combined through two possible mechanisms: electrostatic interactions and covalent binding [12]. In contrast to the complexes formed through electrostatic interactions, covalent conjugates preserve the molecular integrity and improve the functional attributes [13]. One of the most promising methods of protein–polysaccharide covalent bonding with favorable performance is the Maillard reaction. It is a non-enzymatic chemical reaction that occurs spontaneously without the application of chemical reagents [14]. Additionally, as demonstrated in the previous study, the Maillard reaction extent can be controlled easily under wet heating treatment and the reaction time is largely shorter than dry heating [15]. The initial stage of the Maillard reaction involves the condensation between the primary amines of proteins and the reducing-end carbonyl groups of polysaccharides, which is a green modification strategy to make the structure more flexible and improve the functionality of proteins [16]. It has been reported that the Maillard reaction improved the emulsifying properties of the protein via conjugating polysaccharides that enhanced steric stabilization [17]. However, the intermediate and final stage could readily produce a massive number of poorly characterized polymers and brown-colored melanoidins that influence the functionalities of proteins negatively [18]. The Maillard reaction can easily exceed the desired limits, so it is necessary to control the process to avoid the stage later on.

High hydrostatic pressure (HHP) treatment has emerged as a food processing technique that uses a hydrostatic pressure higher than 100 MPa (typically 100–1000 MPa). This technique impacts non-covalent bonds such as electrostatic interactions, hydrogen bonds, hydrophobic interactions, and van der Waals interactions instead of covalent bonds [19]. As a result, it has been regarded as an eco-friendly processing method for protein extraction, physical denaturation, and fortification of functional attributes because of minimal damage to the nutrient composition and sensory qualities of food products [20,21]. In addition, several studies have indicated the influence of HHP combined with moderate heating on the Maillard reaction process [22,23]. Tamaoka, et al. [24] pointed out that during the entire Maillard reaction, the high pressure (50–200 MPa) suppressed the browning reaction instead of the initial condensation reaction. Moreno, et al. [25] mentioned that for buffered and unbuffered media with initial pH ≤ 8.0, the HHP inhibited the different stages of the Maillard reaction differently. Kobayashi, et al. [26] reported that the application of HHP in the thermal treatment of foods can effectively reduce acrylamide content in the Maillard reaction. These studies suggest that HHP can safely suppress the Maillard reaction as a whole process while preserving the quality. However, the emulsifying properties of protein–polysaccharide conjugates prepared by the Maillard reaction under HHP have not been reported. The properties of such conjugates have not been compared with traditional Maillard reaction products and protein-polysaccharide mixtures.

The aim of this study was to investigate the effect of HHP on the Maillard reaction between SPI and SA and to explore the differences in the microstructures and emulsifying properties of the resultant conjugates. An SPI–SA conjugate was prepared via the Maillard reaction under HHP conditions. To contrast the results, an SPI–SA conjugate was additionally prepared via the traditional thermal treatment using a mixture of SPI and SA. The properties of the emulsions stabilized with conjugates (or mixtures) were determined using the particle sizes and zeta potential. Microstructural characteristics of these emulsions were characterized by performing confocal laser scanning microscopy (CLSM).

## 2. Materials and Methods

### 2.1. Materials

SPI (protein content ≥ 90% as per manufacturer) and SA (mean molecular weight = 198 kDa, 98% purity) were purchased from Shanghai Yuanye Bio-Technology Co., Ltd., Shanghai, China. Soy oil was procured from a local grocery and utilized as-received. Ortho-phthaldialdehyde (OPA) and sodium dodecyl sulfate (SDS) were purchased from Sigma Chemical Co., St. Louis, MO, USA. An SDS-polyacrylamide gel electrophoresis (SDS-PAGE) kit, comprising 30% Acr-Bis (29:1), 1.5 M Tris-HCl (pH 8.8), 1.0 M Tris-HCl (pH 6.8), 10% SDS was received from Solarbio Life Sciences, Beijing, China. All other chemicals were of analytic grade.

### 2.2. Preparation of SPI-SA Conjugates and Mixtures

The optimal processing conditions, selected based on single-factor experiments, were as follows: mass ratio of SPI to SA = 1:0.6, buffer pH = 8.0, HHP processing time = 24 h, and pressure = 200 MPa. A mixture of 2% (*w*/*v*) SPI and 1.2% (*w*/*v*) SA powder was dissolved in a sodium phosphate buffer solution (0.05 M, pH 8.0) and subsequently agitated for 2 h at 25 °C before being stored at 4 °C for 24 h to ensure complete hydration. Thereafter, the mixture was divided in three portions. The untreated mixture was labeled SPI-SA-Mix. Another portion was placed in a polyethylene bag twice under vacuum sealed conditions and incubated in an HHP apparatus (HHP-400 MPa, Shenyang Renhe Mechanical & Electrical Engineering Equipment Co., Ltd., Shenyang, China). Pressure treatments were carried out at 200 MPa (60 °C, 24 h) and a cavity medium of deionized water. This sample was labeled SPI–SA–200. The last portion was heated under atmospheric pressure (0.1 MPa) in a thermostat water-bath cauldron at 60 °C for 24 h, and the sample was labeled SPI–SA–0.1. The samples were cooled to room temperature immediately after their treatment and centrifuged (10,000× *g* for 20 min at 4 °C) to remove the insoluble impurities. The supernatants were then freeze-dried for further analysis. 

### 2.3. Measurement of Intermediate Content and Browning Intensity

The intermediate content and the browning intensity were analyzed based on the methods described by Gu, et al. [27] and Chen, et al. [28] respectively. A UV-visible spectrophotometer (Shimadzu UV-2600, Shimadzu Corp., Kyoto, Japan) was employed to evaluate the intermediate content and the browning intensities of the SPI-SA conjugate solutions at 294 nm and 420 nm, respectively. Appropriate dilutions in 0.1% (*w*/*v*) SDS solution were made when required and the solution was used as a blank control.

### 2.4. Determination of the Degree of Grafting (DG)

The DG of the SPI–SA conjugates was evaluated using an OPA reagent based on the reduction of the free amino groups of SPI after the Maillard reaction [29]. The concentration of the sample solutions was diluted to 2 mg/mL, and 200 μL from each diluted solution was incubated with 4 mL of the OPA reagent at 35 °C for 2 min. Employing a UV-visible spectrophotometer, the absorbance of the samples was measured at 340 nm. The blank control was 4 mL of OPA with 200 μL of deionized water. The L-leucine (0–2.0 mM) curve was chosen as the standard to quantify the content of free amino groups. 

The OPA reagent was prepared by adding 40 mg OPA diluted in 1 mL of methanol, 25 mL of 100 mM borax solution, 2.5 mL of 20% (*w*/*w*) SDS solution, and 100 μL β-mercaptoethanol to deionized water, resulting in a final volume of 50 mL. The degree of graft (DG) was calculated using the formula:

(1) $$DG\left(\%\right)=\frac{A_{0}-A_{t}}{A_{0}}\times 100\%,$$

where *A*_0_ and *A_t_* were the total content of free amino groups before and after the reaction, respectively.

### 2.5. Sodium Dodecyl Sulphate–Polyacrylamide gel Electrophoresis (SDS-PAGE)

SDS-PAGE was performed in a discontinuous buffer system based on the Laemmli [30] method. The molecular weight range of the protein marker was 11–180 kDa. A 12% separating gel and a 5% stacking gel were used. The samples were dissolved in 0.15 M Tris-HCl buffer (pH 6.8) comprising 20 mL glycerol, 4.1 g SDS, 2 mL β-mercaptoethanol (ME), and 0.02 g bromophenol blue with deionized water to a final volume of 100 mL and then boiled for 5 min. The current in the stacking gel was set to 80 V, and the voltage was increased to 120 V when the samples entered the separation gel. After the electrophoresis, the gel was dyed for protein with 0.1% (*w*/*v*) Coomassie Brilliant Blue R-250 in 50% (*v*/*v*) methanol and 6.8% (*v*/*v*) glacial acetic acid and then decolorized by mixing with methanol (50 mL) and glacial acetic acid (75 mL) to a final volume of 1000 mL using deionized water.

### 2.6. Circular Dichroism (CD) Spectroscopy

CD spectroscopy was performed based on the procedure of Fu, et al. [31] with modifications. Changes in the secondary structure of the protein were monitored using a CD spectrometer (J-815, JASCO, Tokyo, Japan). The samples were diluted to a protein concentration of 0.1 mg/mL using a phosphate buffer (0.01 M, pH 7.0), and a blank phosphate buffer was used for the determination. The parameters were set as follows: scanning range = 190–250 nm, temperature = 25 °C, quartz cell path length = 0.1 cm, band width = 1 nm, scan rate = 50 nm/min, and scanning interval = 0.5 nm. The conformational changes in the secondary structures, including α-helix, β-sheet, β-turn, and random coil, were deduced using the computer program CDPro (http://dichroweb.cryst.bbk.ac.uk/html/process.shtml, accessed on 5 January 2021) [32].

### 2.7. Intrinsic Fluorescence Spectroscopy

The intrinsic fluorescence spectra of the prepared samples were measured with a fluorescence spectrophotometer (F-4500, Hitachi, Ltd., Tokyo, Japan). The samples were dispersed in phosphate buffer (0.01 M, pH 7.0) to obtain a final solution of 1 mg/mL in protein. Subsequently, 1 mL of the diluted solution was excited at 295 nm in a four-way cuvette (5 nm slit width), and the emission spectra were measured from 310 to 450 nm at a scanning rate of 1200 nm/min.

### 2.8. Emulsifying Property Analysis

The emulsifying activity index (EAI) and emulsion stability index (ESI) were determined according to the procedure described by Zhu, et al. [33] with modifications. For the emulsion formation, 5 mL of soy oil and 15 mL of the sample were homogenized in a phosphate buffer solution (0.1 M, pH 7.0, 1 mg/mL protein) with a digital homogenizer (Ultra-Turrax T50, IKA, Staufen, Germany) for 5 min at 10,000 rpm. A small amount (50 µL) of the homogenized emulsion were extracted from the bottom after 0 min and 10 min and diluted in 0.1% (*w*/*v*) SDS solution (5 mL). The absorbance of the diluted emulsion samples was measured at 500 nm using a UV-visible spectrophotometer (Shimadzu UV-2600, Shimadzu Corp., Kyoto, Japan). EAI (m^2^/g) and ESI (min) values were calculated as follows:

(2) $$EAI=\frac{2\times 2.303\times DF\times A_{0}}{C\cdot \varphi \cdot \theta \times 10^{4}},$$

(3) $$ESI=\frac{A_{0}}{A_{0}-A_{10}}\times 10,$$

where *A*_0_ and *A*_10_ are the absorbances of the diluted emulsions at 0 min and 10 min, respectively, *DF* is the dilution factor (100), *C* is the concentration of protein (g/mL) before emulsification, *φ* is the proportion of the oil phase (0.25), and *θ* is the optical path (1 cm).

### 2.9. Emulsion Particle Size

The volume-averaged particle sizes and particle size distributions for the prepared emulsions were measured with a laser light scattering instrument (Zetasizer Nano, Malvern Instruments Ltd., Worcestershire, UK). The particle sizes and the particle size distributions were expressed in terms of the mean diameter (size, nm) and polydispersity index (PDI). The measurements were performed thrice.

### 2.10. Emulsion Zeta Potential

The emulsions were injected into the capillary absorption cuvette, and the zeta potential was monitored using a zeta potentiometer (ZetasizerNano, Malvern Instruments Ltd., Worcestershire, UK). The measurements were performed thrice.

### 2.11. CLSM

Microscopic characteristics, such as the sizes and distributions of oil droplets and proteins in the emulsions were determined by performing CLSM (Leica TCS SP2 CLSM, Leica Microsystems Inc., Heidelberg, Germany). Nile Red staining solution (25 μL; Nile Red dissolved in anhydrous ethanol (1 mg/mL) for oil droplet staining; excitation at 488 nm) and 20 μL of Nile Blue staining solution (Nile Blue dissolved in isopropanol (10 mg/mL) for protein staining; excitation at 633 nm) were added to 1 mL of emulsion and then shaken, mixed, and stored in the shade for 0.5 h. The stained emulsion (5 µL) was placed on a concave microscope slide, which was then covered with cover-slips without air bubbles. Subsequently, CLSM was performed with a 100× objective lens (oil) in a short time.

### 2.12. Statistical Analysis

Each experiment was repeated thrice. The results were evaluated as mean ± standard deviation (SD). The analysis of variance (ANOVA) was conducted using SPSS software (SPSS 17.0 for Windows, SPSS Inc., Chicago, IL, USA). The statistical significance was set at *p* < 0.05.

## 3. Results and Discussion

### 3.1. Effect of HHP on the Maillard Reaction between SPI and SA

The measurements of the intermediate content, browning intensity, and DG are commonly used as indicators of the extent of the Maillard reaction. During the Maillard reaction, the reducing sugars are cleaved to produce small colorless aldehydes and ketones, which exhibit the maximum absorption at 294 nm [34]. Moreover, as the Maillard reaction progresses to an advanced stage, brown matter is produced and absorbed at 420 nm [35]. Therefore, the absorbances at 294 nm and 420 nm are generally chosen to represent the intermediate content and browning intensities, respectively. As shown in Table 1, the HHP treatment significantly reduced the absorbances at 294 nm and 420 nm (*p* < 0.05) compared to those of the SPI–SA–0.1 conjugate, indicating its inhibitory effect on the SPI–SA Maillard reaction. Isaacs and Coulson [36] and Martínez, et al. [37] found that the intermediate and advanced stages of the Maillard reaction were suppressed, which indicated a retardation of the degradation of the Amadori rearrangement products.

**Figure 1:**
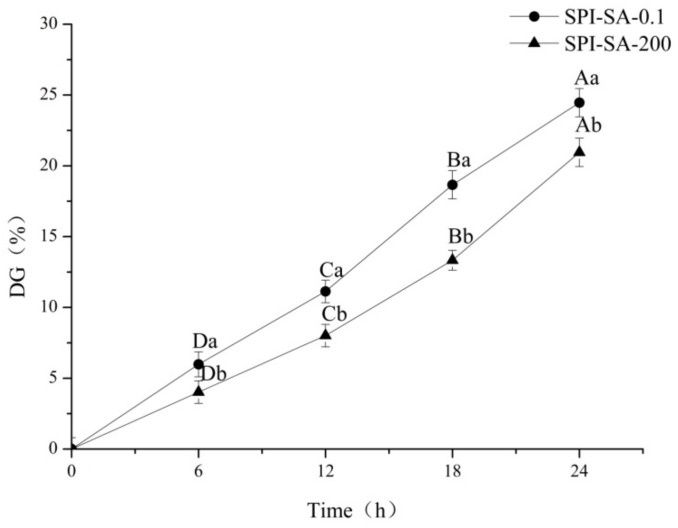
The DG values of the SPI–SA–0.1 and SPI–SA–200 conjugates in the graft reaction process. The lowercase letters represent a comparison between different samples at the same time; the uppercase letters represent a comparison between different instants of the same sample; the letters a–b and A–D indicate decreasing order of magnitudes (*p* < 0.05).

The evolution of the DG values of the SPI–SA–0.1 and SPI–SA–200 conjugates is shown in Figure 1. When the reaction time was extended to 24 h, a DG value of 20.95% was consistently observed in case of the Maillard reaction at 200 MPa, whereas a DG value of 29.57% was observed in case of the heating process at 0.1 MPa. The DG percentages of the SPI–SA–200 conjugate (prepared via HHP treatment) were significantly lower in comparison with those of the SPI–SA–0.1 conjugate. This is because an increase in pressure can retard the reaction rate, which depends ona positive activation volume of the Maillard reaction at a constant temperature [38]. The complexity of the Maillard reaction signals that it occurs through multiple reaction pathways. However, previous studies have reported that several pathways have a positive activation volume, including the decomposition of the Amadori rearrangement products, which is one of the most important pathways [39,40]. Therefore, it can be summarized that the Maillard reaction generally regarded as a whole has a positive activation volume, and is suppressed by high pressure [41].

### 3.2. SDS-PAGE Analysis

**Figure 2:**
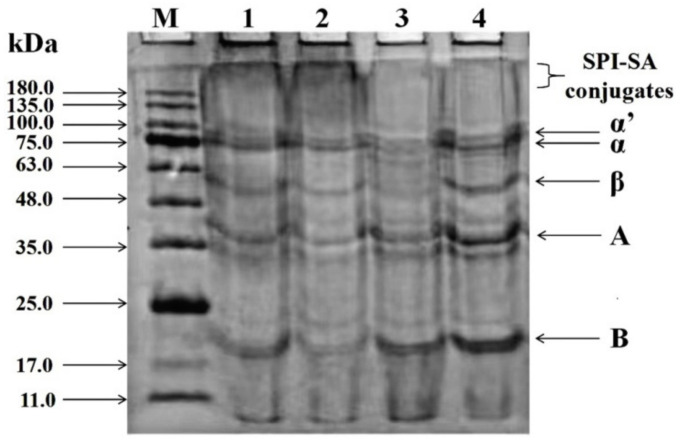
The SDS-PAGE patterns of molecular weight markers (lane M) and the SPI-SA-0.1 (lane 2), SPI–SA–200 (lane 1), SPI–SA–Mix (lane 3), and SPI (lane 4) samples; β-conglycinin (7s): α’, α, and β, glycinin (11s): A and B.

SDS-PAGE was performed to verify the presence of covalent linkages between SPI and SA and to determine the distribution and variation of sub-units. Figure 2 displays the protein bands of SPI–SA–200 (lane 1), SPI–SA–0.1 (lane 2), SPI–SA mixture (lane 3), and SPI (lane 4). SPI (lane 4) exhibited five pronounced bands corresponding to sub-units of β-conglycinin (α’, α, and β) and glycinin (A and B) [42].

The SPI–SA conjugates (lanes 1 and 2) exhibited different degrees of decline in the band color intensities, and new bands with significantly higher molecular weights were observed on the top of the gel. This revealed that SPI exhibited a noticeable shift in its molar mass distribution toward higher molecular weights, and aggregates with high molecular weights were formed [43]. Thus, it was confirmed that the SPI molecules were covalently bonded with the SA molecules. This result was in accordance with the study of Chen et al. on the polymerization between whey protein isolate and gum acacia carried out by performing SDS-PAGE [28]. In addition, the change in the protein band of SPI–SA–0.1 (lane 2) was more pronounced than that of SPI–SA–200 (lane 1). Hence, it was concluded that the HHP (200 MPa) decelerated the Maillard reaction, which was in accordance with the intermediate content, browning intensity, and DG results. The results for the SPI–SA mixture (lane 3) additionally revealed that the band colors for the sub-units of β-conglycinin were shallow or approximately pale. This could be a result of non-covalent interactions, since protein molecules, which were wrapped in polysaccharide molecules, interacted weakly with SDS.

### 3.3. Circular Dichroism (CD) Spectral Analysis

The far-UV CD spectrum (spectrum in Figure S1) was used to analyze the secondary structures of SPI, SPI–SA mixture, SPI–SA–0.1, and SP-SA-200 (Table 2). The SPI–SA mixture exhibited low α-helix and β-turn content and significantly high β-sheet and random coil content. These results are similar to those reported by Liu, et al. [44], wherein the electrostatic interaction between proteins and polysaccharides affected the secondary structures of the proteins. Additionally, the α-helix and β-sheet content of SPI–SA–0.1 were lower than those of SPI, whereas the β-turn and random coil content of SPI–SA–0.1 were higher than those of SPI. This variation can be attributed to the attachment of the SA molecules to the SPI molecules via the Maillard reaction, which leads to instabilities, decomposition of the α-helix structure, and a disorderly spatial structure [45]. The SPI–SA–200 conjugate exhibited low α-helix and high random coil values in comparison with those of SPI–SA–0.1. This indicates that the HHP treatment could further promote the unfolding of the SPI structure and the exposure of the internal groups [46]. As the stability of the secondary structures is maintained by various types of hydrogen bonds, the HHP treatment modifies the structures by destroying the hydrogen bonds of α-helices and β-sheets and forming excess β-turns and random coils [37]. These changes in the secondary structure could enhance the protein flexibility and the adsorption at the oil-water interface, resulting in improved emulsifying properties.

### 3.4. Intrinsic Fluorescence Spectroscopy Analysis

**Figure 3:**
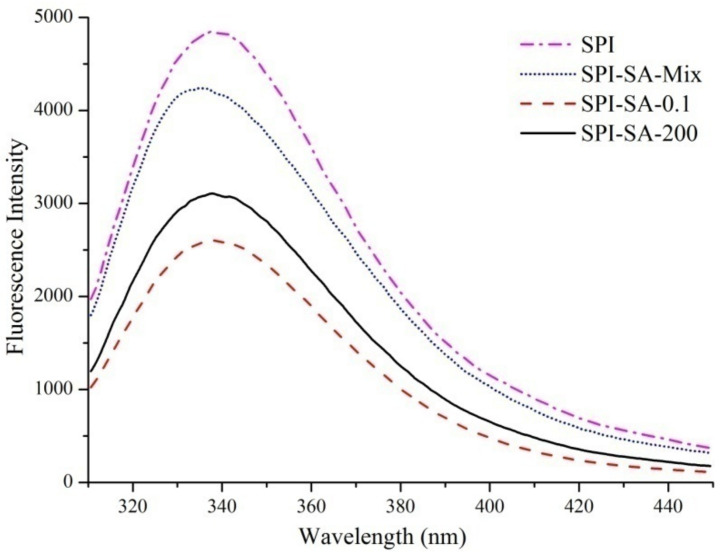
The intrinsic fluorescence spectra of the SPI, SPI–SA mixture, SPI–SA–0.1, and SPI–SA–200 samples.

Intrinsic fluorescence spectroscopy was used to obtain structural information of the protein. The measurements of changes in the spectra of SPI, SPI-SA mixture and conjugates are present in Figure 3. The fluorophore of protein molecules was mainly attributed to the Trp, Tyr and Phe residues, particularly the Trp residue whose fluorescence intensity was frequently applied to analyze the changes in protein conformation [28]. When excited at 290 nm, SPI exhibited a fluorescence emission maximum (λ_max_) at 337.8 nm, while a blue-shift of the λ_max_ at 335.0 nm appeared in SPI–SA mixture spectra. The red-shift of λ_max_ to different degrees was observed for SPI–SA conjugates, with SPI–SA–0.1 at 339.8 nm and SPI–SA–200 at 338.0 nm. Presumably, the Maillard reaction altered the local microenvironment of the Trp residue to a greater polarity, improving the hydrophilicity of the protein [47]. On the contrary, the fluorescence intensity of conjugates decreased, indicating that non-fluorescent polysaccharide molecules with a larger steric hindrance shielded the Trp fluorophore and reduced the fluorescence intensity of protein molecules [18]. Similarly, Feng et al. revealed that the SPI–polysaccharides mixtures showed lower intensity values, and conjugates showed the lowest intensity value [48]. The fluorescence intensity of conjugates under 200 MPa was stronger than 0.1 MPa due to the less non-fluorescent polysaccharide attached to the protein, which proves the retarding effect of HHP on the Maillard reaction.

### 3.5. Emulsifying Properties

#### 3.5.1. Emulsifying Activity and Stability

**Figure 4:**
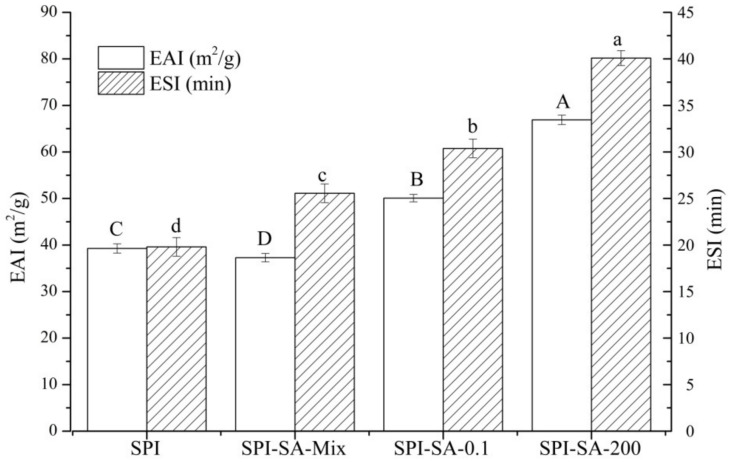
The emulsifying properties of the SPI, SPI–SA mixture, SPI–SA–0.1, and SPI–SA–200 samples. The lowercase and uppercase letters represent the comparison of ESI and EAI between samples, respectively. The letters a–d and A–D indicate decreasing orders of magnitude (*p* < 0.05).

As is shown in Figure 4, significant improvements in the EAI and ESI can be achieved by combining HHP with the Maillard reaction. The EAI of SPI significantly decreased upon physical mixing with SA. It is deduced that the high solution viscosity of SPI is caused by the presence of SA and the electrostatic interaction between the two separate biopolymers, which weakens the interfacial properties of SPI [49]. The SPI–SA–0.1 and SPI–SA–200 conjugates exhibited enhancements of 30.56% and 70.36% in their EAI values, respectively, in comparison with that of SPI. This result is attributed to the hydrophilic polysaccharide connected to the protein, which combines the significant adsorbability of the protein at the oil-water interface with the solvation of the polysaccharides in the aqueous medium, resulting in an increased interfacial activity [50]. Additionally, the ESI was significantly (*p* < 0.05) enhanced by either the covalent or non-covalent bonding between the SPI and the reducing sugars. The SPI–SA–0.1 and SPI–SA–200 conjugates exhibited enhancements of 18.86% and 56.85% in the ESI values, respectively, in comparison with that of the SPI–SA mixture. This could be attributed to the covalent binding of SA to the protein, which led to the formation of a viscoelastic layer and an enhancement in the steric stabilization, resulting in the prevention of creaming, flocculation, and coalescence [51]. The EAI and ESI values of SPI–SA–200 were significantly higher than those of SPI–SA–0.1 (*p* < 0.05), which further facilitated the unfolding of the SPI structure at HHP, resulting in an increased molecular flexibility. Moreover, the HHP treatment improved the hydrophilicity of SPI and exposed the hydrophobic groups to improve the amphiphilic balance [52]. In addition, the HHP treatment improved the emulsifying ability by preventing the SPI molecules from interacting with a significant number of polysaccharide molecules that could affect the interfacial activity and the internal structural stretching of the proteins [53].

#### 3.5.2. Particle Sizes of Emulsions

**Figure 5:**
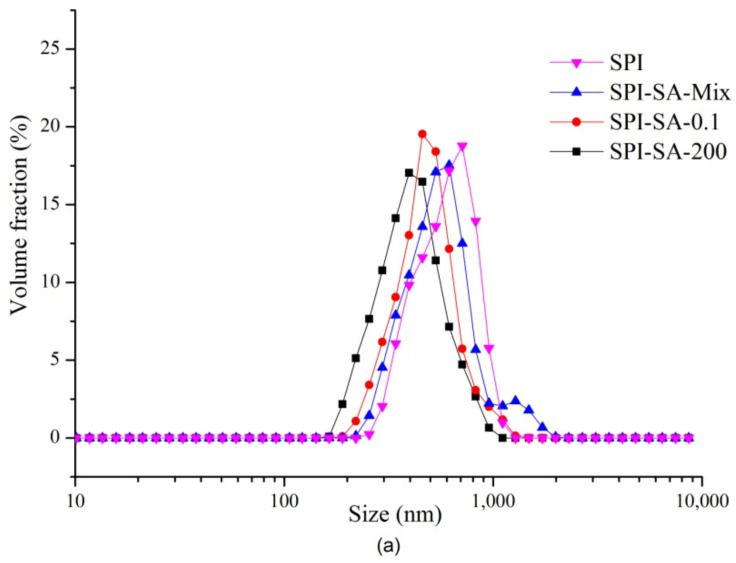

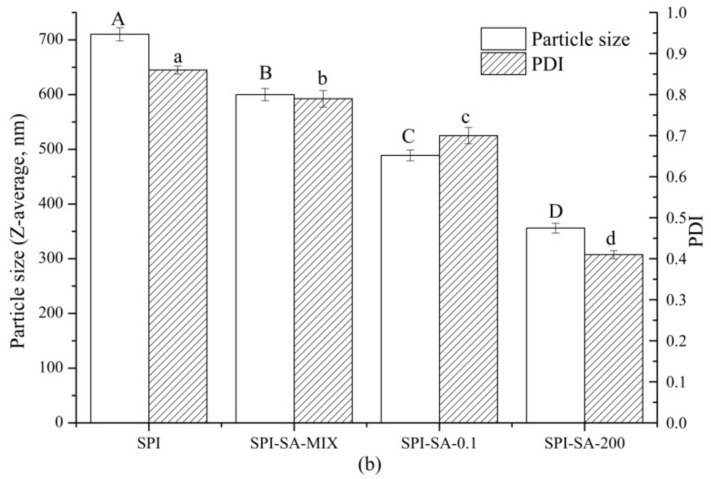
The particle size distributions (**a**) and average particle sizes (**b**) for the emulsions stabilized by the SPI, SPI–SA mixture, SPI–SA–0.1, and SPI–SA–200 samples. The lowercase and uppercase letters represent comparisons of the Z-average particle sizes and PDI values between emulsion samples. The letters a–d and A–D indicate decreasing orders of magnitude (*p* < 0.05).

The average particle size and distribution data of the emulsions stabilized by the SPI-SA mixture and the conjugates are used to evaluate the emulsifying properties (Figure 5a,b). Both the SPI-SA conjugates formed emulsions with monomodal particle distributions, while the emulsion of the SPI–SA mixture exhibited a bimodal particle distribution, and the size and PDI of the SPI–SA–0.1 and SPI–SA–200 conjugates were significantly reduced in comparison with those of the mixture. These results suggest that the conjugates exhibit significant improvements in terms of the average particle size and PDI values compared to those of the non-conjugated SPI–SA mixture. This result is in agreement with earlier studies on fenugreek gum and soy–whey protein isolates [16]. The emulsion stabilized by the SPI–SA–200 conjugate exhibited lower average particle size (356 nm) and PDI (0.41) values in comparison with those of the SPI–SA–0.1 conjugate (average particle size = 489 nm and PDI = 0.7). Therefore, combining the HHP treatment with the Maillard reaction resulted in a beneficial and synergistic formation of a homogeneous emulsion, which could inhibit protein aggregation.

#### 3.5.3. Zeta Potential of Emulsions

**Figure 6:**
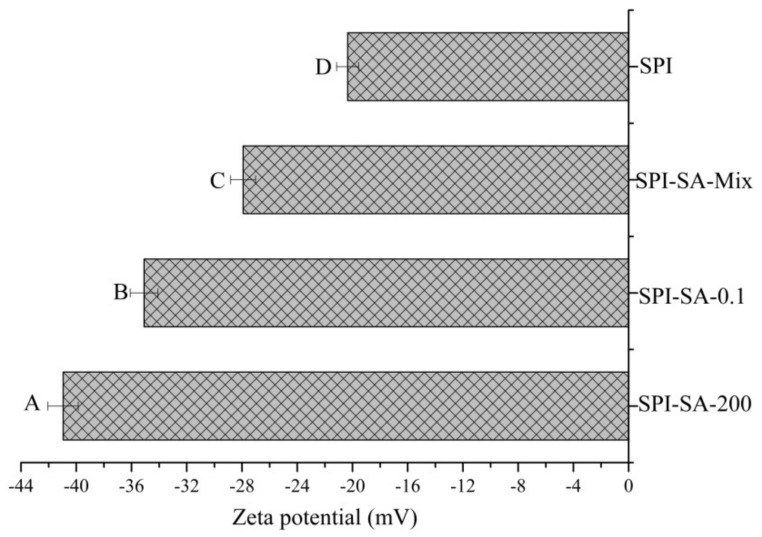
The zeta potential of the emulsions stabilized by the SPI, SPI–SA mixture, SPI–SA-0.1, and SPI–SA–200 samples. The letters A–D indicate decreasing orders of magnitude (*p* < 0.05).

The zeta-potential of the emulsions exhibited the charged nature of the droplet and could reflect the interactions between the emulsion droplets, thus indicating the stability of the emulsions [54]. Figure 6 presents the zeta potentials of the SPI–SA mixture and the conjugates. The zeta potential of the emulsions stabilized by SPI (at pH 7.0) was −20.15 mV. A negative zeta potential of −27.92 mV was observed for the SPI-SA mixture. Negativezeta potentials were additionally observed for the SPI–SA conjugates. The zeta potentials of the conjugates exhibited significant enhancements, which could be attributed to the diminution of the applicable free amino groups (NH_4_^+^) after conjugation [55]. Similar results were previously reported for the conjugation of whey protein with rhamnolipid [56]. In particular, the negative zeta potential enhancement was most pronounced in the case of the HHP treatment (200 MPa), which could be attributed to the formation of negatively charged intermediaries during the Maillard reaction and the exposure of a higher number of charged groups in SPI during the HHP treatment [8]. A higher negative zeta potential could prevent the coalescence of emulsions by enhancing the electrostatic repulsion between the droplets.

#### 3.5.4. Emulsion Microstructure

**Figure 7:**
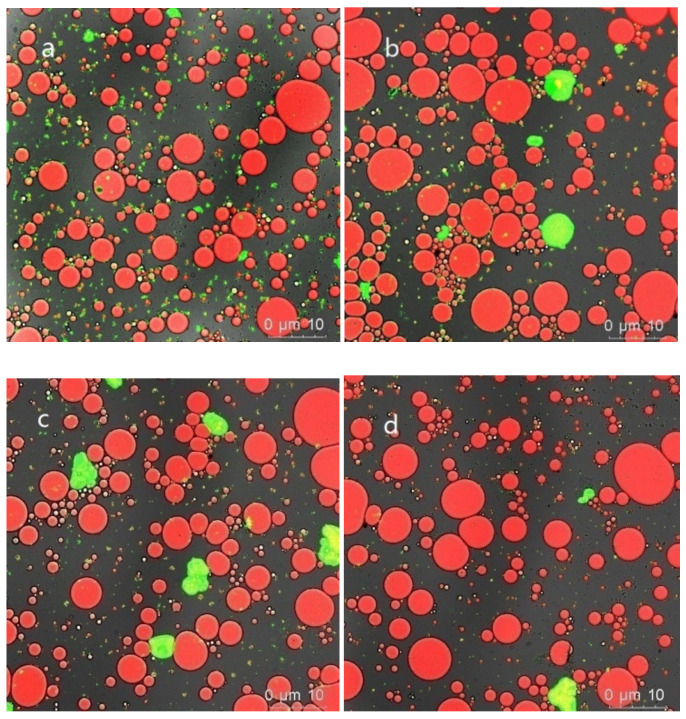
The CLSM images of the microstructures of the emulsions stabilized by the SPI (**a**), SPI–SA mixture (**b**), SPI–SA–0.1 (**c**), and SPI–SA–200 (**d**) samples.

The CLSM images revealed the microstructures of the emulsions stained with Nile red (oil droplet stain, red) and Nile blue (protein stain, green). As shown in Figure 7, the oil droplets of the SPI emulsion were large-sized and in a flocculated state. The size of oil droplets of the SPI–SA–Mix emulsion were smaller but *the droplets were not evenly distributed*. In comparison, the conjugate-stabilized emulsions did not exhibit a flocculated state. Additionally, they exhibited relatively homogeneous and smaller droplet sizes in comparison with those of the SPI and SPI–SA–Mix emulsions. Furthermore, the emulsion stabilized with SPI–SA–200 exhibited higher homogeneity in the droplet sizes compared to that of the emulsion stabilized with SPI–SA–0.1, indicating that the HHP treatment resulted in a significantly improved emulsion stability. These results were consistent with those of the zeta potential and particle size.

## 4. Conclusions

In this study, we demonstrated the effect of HHP treatment combined with the Maillard reaction on the structural and emulsifying properties of SPI. Intermediates, browning intensity, and DG% values revealed that HHP reduced the extent of the Maillard reaction. The secondary structure of SPI-SA conjugate was also altered at 200 MPa, in terms of the unfolding molecular structure and the disordered spatial structure. The emulsifying properties of SPI–SA–200 were effectively enhanced compared with that of the SPI–SA–0.1 and SPI–SA mixture; the particle size, zeta potential and CLSM images also indicated that the emulsion stability of SPI–SA 200 was improved. The results suggested that using HHP hindered the Maillard reaction and might be a practical means to improve the emulsifying properties of SPI and a potential method applied in the food industry.

## Figures and Tables

**Table 1:** The evaluation of the intermediate content and browning intensities of the SPI, SPI–SA mixture, SPI–SA–0.1 (0.1 MPa), and SPI–SA–200 (200 MPa) samples. The conjugates were prepared at 60 °C.

Samples	SPI	SPI-SA-Mix	SPI-SA-0.1	SPI-SA-200
A_294_	0.09 ± 0.013 ^d^	0.12 ± 0.007 ^c^	0.82 ± 0.03 ^a^	0.54 ± 0.01 ^b^
A_420_	0.02 ± 0.003 ^d^	0.03 ± 0.005 ^c^	0.17 ± 0.008 ^a^	0.10 ± 0.003 ^b^

The values represent means ± standard deviations; The ranges ^(a–d)^ represent the row-wise decreasing magnitudes of the absorbances (*p* < 0.05).

**Table 2:** The secondary structural compositions (%) of the SPI, SPI–SA–Mix, SPI–SA–0.1, and SPI–SA–200 samples.

Sample	α-Helix (%)	β-Sheet (%)	β-Turns (%)	Random Coil (%)
SPI	14.60 ^a^	27.03 ^b^	10.22 ^c^	48.15 ^d^
SPI-SA-Mix	13.10 ^b^	27.44 ^a^	9.58 ^d^	49.88 ^c^
SPI-SA-0.1	11.51 ^c^	26.09 ^c^	11.96 ^a^	50.44 ^b^
SPI-SA-200	9.08 ^d^	25.32 ^d^	10.98 ^b^	54.62 ^a^

The ranges ^(a–d)^ represent the columnwise decreasing magnitudes of the percentages (*p* < 0.05).

## Data Availability

The data that support the findings of this study are available on request from the corresponding author. The data are not publicly available due to privacy or ethical restrictions.

## Supplementary Materials

The following are available online at https://www.mdpi.com/article/10.3390/foods10112829/s1, Figure S1: Far UV CD spectrum of SPI, SPI–SA mixture, SPI–SA–0.1, and SP–SA–200.
