# Supplementary material for: Structural and Emulsifying Properties of Soybean Protein Isolate–Sodium Alginate Conjugates under High Hydrostatic Pressure
Source: Foods. 2021 Nov 17;10(11):2829. doi: 10.3390/foods10112829 (PMC8620206; doi:10.3390/foods10112829)
Supplement: Supplementary file 1 [file foods-10-02829-s001.zip › foods-1415483-supplementary.pdf]

Supplementary Materials:

1. The single-factor experiment:

The single-factor experiment of the mass ratio: SA and SPI were dissolved in phosphate buffer solution (0.05 mol/L, pH 8) at the mass ratios of 0.4:1, 0.6:1, 0.8:1, 1:1, and 1:1.2, respectively, and then place all samples in the HHP equipment, the processing pressures: 100 MPa, 200 MPa, and 300 MPa, respectively at 60℃, processing time= 24 h. The degree of grafting (DG) and emulsifying activity of the products were measured, and the 2% (W/V) SPI was used as a comparison.

The single-factor experiment of the HHP processing time: SA and SPI were dissolved in phosphate buffer solution (0.05 mol/L, pH 8) at the mass ratios of 0.6:1, and then place the sample in the HHP equipment, the processing pressures: 100 MPa, 200 MPa, and 300 MPa, respectively at 60℃. The high pressure processing times was 12 h、18 h、24 h、30 h、36 h、42 h, respectively. The degree of grafting (DG) and emulsifying activity of the products were measured, and the 2% (W/V) SPI was used as a comparison.

The single-factor experiment of the buffer pH: SA and SPI were dissolved in phosphate buffer solution (0.05 mol/L, pH 7、pH 7.5、pH 8、pH 8.5、pH 9) at the mass ratios of 0.6:1, and then place the sample in the HHP equipment, the processing pressures: 100 MPa, 200 MPa, and 300 MPa, respectively at 60℃. The high pressure processing times was 24 h. The degree of grafting (DG) and emulsifying activity of the products were measured, and the 2% (W/V) SPI was used as a comparison.

The optimal processing conditions, selected based on single-factor experiments, were as follows: mass ratio of SPI to SA = 1:0.6, buffer pH = 8.0, HHP processing time = 24 h, and pressure = 200 MPa.

2. The far-UV CD spectrum:

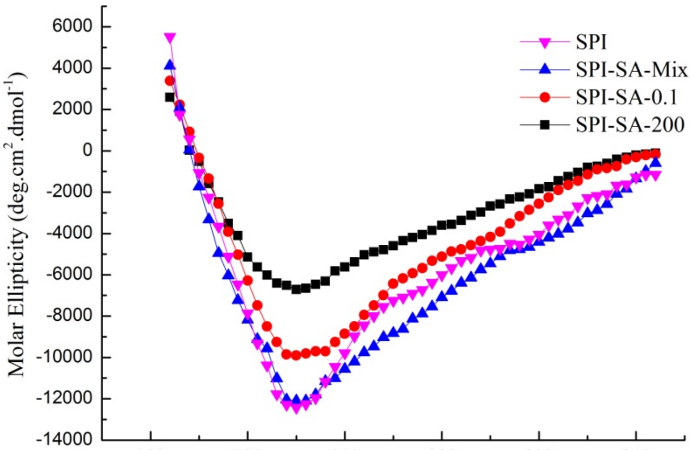

Figure S1. Far UV CD spectrum of SPI, SPI-SA mixture, SPI-SA-0.1, and SP-SA-200.
